# The Practice of Shaking in Disciplining Young Children in Lower-Income Communities of Bangladesh: Cross-Sectional Exploratory Study

**DOI:** 10.2196/64474

**Published:** 2025-10-14

**Authors:** Fahmida Tofail

**Affiliations:** 1 International Centre for Diarrhoeal Disease Research, Bangladesh Dhaka Bangladesh

**Keywords:** infant shaking, behavior, depression, urban slum, Matlab, Dhaka, Bangladesh

## Abstract

**Background:**

Disciplining a child is a complex, multidimensional aspect of parenting, involving emotional guidance, behavioral correction, and consistent communication. Disciplinary practices applied to children may vary due to social, cultural, and geographic contexts. Among these practices, child shaking and its potentially severe consequences, including traumatic brain injury and long-term neurodevelopmental impairments, are more frequently documented in high-income countries. Although child shaking also occurs in low- and middle-income countries, particularly among infants, where it is primarily triggered by persistent crying, it is rarely reported in formal studies. Moreover, primary caregivers, particularly mothers, may not perceive shaking as a form of punitive discipline, and maternal depression is often associated with their caregiving attitude toward children. Furthermore, notable functional variations are also observed in the parenting practices of low-income urban and rural mothers, often influenced by divergent socioeconomic and environmental factors.

**Objective:**

This study aims to explore caregivers’ use of shaking as a disciplinary practice with young children in lower-income urban and rural communities in Bangladesh and identify the underlying factors associated with this behavior.

**Methods:**

This was a cross-sectional study in which we randomly interviewed 800 caregivers of children (aged <2 y) admitted to the hospitals of the International Centre for Diarrhoeal Disease Research, Bangladesh, in urban Dhaka (n=520, 65%) and rural Matlab (n=280, 35%). We collected detailed information on how they disciplined their young children (index children), with particular emphasis on shaking behavior. The questionnaires also explored perceived causes and common beliefs related to child shaking, in addition to collecting information on sociodemographic characteristics and caregivers’ depression. All analyses were conducted using SPSS software.

**Results:**

Child shaking was higher in lower-income communities of Dhaka compared to rural Matlab (n=265, 51% vs n=52, 19%; *P*<.001). Mothers, as the primary caregivers, mostly used shaking as a disciplinary practice with their young children (n=491, 61%). Overall, 27% (n=214) of primary caregivers reported ever shaking a child below 2 years; among these, 62% (n=133) reported shaking at least 3 to 5 times. Crying and fussing were the main triggering factors (Dhaka: n=107, 62% vs Matlab: n=26, 67%) for shaking behavior, particularly in infants aged younger than 6 months. Approximately 56.6% (n=99) of the mothers in Dhaka and 28.2% (n=11) of the mothers in Matlab believed that shaking was either definitely or probably harmless. Maternal depression was significantly correlated (adjusted odds ratio 1.03, 95% CI 1.01-1.04; *P*=.001) with shaking practices used by mothers.

**Conclusions:**

Shaking young children as a form of discipline is quite prevalent in lower-income communities of Bangladesh, where poverty and other stressors are common. Caregivers are not fully aware of the harmful side effects of infant shaking. Positive parenting programs need to be strengthened to reduce these harmful behaviors toward children to support their developmental potential.

## Introduction

### Background

Shaking infants, commonly reported in Western countries, is a practice used by caregivers as a form of physical discipline or parenting for young infants and children. However, shaking can be the primary risk behavior for shaken baby syndrome (SBS) or abusive head trauma (AHT). Shaking infants or young children can cause a range of harms, from mild and transient effects to severe and potentially life-threatening injuries. Studies estimate that 10% to 12% of the infant deaths related to child abuse are attributable to SBS, with approximately 1 in 4 deaths due to grave injuries [1,2]. SBS is a severe form of child abuse caused by violent shaking, leading to brain injuries, such as subdural hematomas, diffuse axonal injury, and retinal hemorrhages, as well as fractures of the long bones or ribs, even when there might be little or no evidence of trauma [3]. Infants are particularly susceptible to injuries due to their anatomical and physiological characteristics, including a heavy head, weak neck muscles, a soft and rapidly growing brain, and a thin skull wall. With less control over their head and neck and limited mobility, they face an increased risk of permanent injuries. Globally, shaking is recognized as a common disciplinary act for children. In the United States, 2.6% of the parents of children aged younger than 2 years acknowledge shaking their child as an act of “discipline.” This indicates that the ratio of reported shaking to severe or fatal cases of SBS or AHT is 152:1 [4]. When data from 60 countries were analyzed, it was found to be 33% more common [5]. Despite the mortality rate for SBS and AHT ranging from 13% to 30% in high-income countries such as the United States [6-8] and Canada [9], its prevalence and long-term impacts in low- and middle-income countries (LMICs) remain underexplored. In Japan, the prevalence of self-reported shaking behavior at 1 month post partum was 16.8%, with caregivers continuing this behavior until the infants were aged 6 years [10]. Although several systematic reviews have been conducted on the impact and prevalence of SBS injuries in high-income countries, evidence from LMICs is unavailable. A parallel survey was conducted on parental child-discipline practices in 6 different countries that included a high-income country (the United States), upper–middle-income countries (Brazil and Chile), and lower–middle-income countries (Egypt, Philippines, and India). The study showed that almost half of the surveyed communities practiced shaking as a typical caregiving act, and more than 20% indicated using shaking to discipline their children before they were aged 2 years [11].

The consequences of such harsh punishments, particularly child shaking, are not well understood in LMICs, where poverty and other psychosocial stressors, such as violence, aggression, and caregivers’ depression, are quite common [12]. The long-term public health implications of child shaking remain largely unknown, emerging evidence suggests that it may contribute to the prevalence of learning disabilities and intellectual impairments in these settings [13]. Social and economic adversities may amplify the risks associated with such harsh caregiving practices, underscoring the need for further research and culturally appropriate interventions.

Common risk factors for the perpetrators of child shaking include an inability to cope with stress, poor impulse control, unrealistic child-rearing expectations, and rigid attitudes. In addition, caring for premature or high-needs infants may lead to heightened frustration, reduced tolerance, and resentment, increasing the likelihood of harmful responses [14]. In these situations, parents and caregivers often consider shaking to be less harmful than spanking. Environmental factors, such as low socioeconomic status, poverty, unemployment, low education, unsafe neighborhoods, poor prenatal care, single marital status, frequent moves, and the lack of social support, further compound the risk of infant shaking [15].

The long-term consequences of SBS are severe. Children exposed to SBS before the age of 2 years have exhibited developmental delays in 11% to 47% of the cases by the age of 5 years [16].

### Objectives

Bangladesh, a densely populated, low-income country with more than 165 million people, is facing unique challenges in addressing harmful caregiving practices. In this study, we aimed to explore the descriptive epidemiology of caregiver-executed shaking behavior among young children residing in lower-income, resource-constrained urban and rural communities of Bangladesh.

The specific objectives of this study were to (1) estimate the overall burden of shaking in children aged between 0 and 24 months, (2) identify the common triggering factors for such behavior, and (3) understand caregivers’ descriptions of the act of shaking and their awareness of its potentially harmful effects.

## Methods

### Design

This cross-sectional survey was conducted among the mothers and other caregivers of children aged younger than 2 years who were admitted to the 2 big charity hospitals of the International Centre for Diarrhoeal Disease Research, Bangladesh. The 2 hospitals are situated in urban Dhaka and rural Matlab. Both hospitals primarily serve the economically disadvantaged surrounding communities. This study was conducted over 7 months, from June to December 2009. Children aged younger than 2 years with less severe diarrhea were enrolled in this study. We excluded all children who were critically ill (eg, children experiencing convulsions and those with tuberculosis, encephalitis, and meningitis) and those with obvious physical congenital anomalies or developmental abnormalities. Due to the variation in patient flow between the 2 hospitals, 2 interviewers at the Dhaka Hospital recruited up to 6 mother-child dyads per day (n=520), while one interviewer at Matlab Hospital recruited up to 3 dyads per day (n=280).

### Measures

A questionnaire was adapted that included both open- and closed-ended items, designed to capture the experiences of caregivers with child discipline, specifically focusing on shaking behavior and their associated mental states, such as the extent of anger or frustration during these incidents.

A structured socioeconomic and demographic questionnaire was used to collect information on the number of family members, parental education and occupation, number of children aged younger than 5 years in the family, and the birth order of the index child. In addition, participants were asked about household income and expenditure balances over the previous months. The financial status of the families was classified into 4 categories: constant deficits, occasional deficits, balanced income, and surplus (excess of income over expenditure). The occupation categories were classified into 3 groups based on skill level and employment regularity: unskilled (eg, unemployed individuals and day laborers), low-skilled and irregular (eg, mechanics, electricians, and rickshaw pullers), and high-skilled job categories.

Questions on child discipline practices assessed whether the caregivers had ever disciplined their child and, if so, who else had been involved in the disciplinary process and what methods were used. *To capture caregiver-reported child-shaking behavior,* 5 *items were adapted* from the “Parent-Child Conflict Tactics Scale (CTSPC)” [17]. CTSPC includes subscales assessing various dimensions of the parent-child relationship, such as communication, power dynamics, discipline strategies, emotional expression, and relationship quality. Items are typically rated on a Likert-type scale (eg, from “never” to “always”), and subscale scores are calculated by summing the individual item responses.

The selected questions from this questionnaire included: (1) “Has anyone in the household ever shaken the child to discipline?” (2) “If yes, how many times in the past year or since birth was the child shaken?” and (3) “What was the mental status of the caregiver or others while they shook the baby?” Two additional items from a previous study were incorporated to assess contextual factors, assessed by the question, “What was the child doing right before you shook them?” and awareness of the potential consequences of shaking, assessed by the question, “Thinking about when you shook your child, did you think you might have hurt the child?” These items had previously been field-tested in the United States and other countries [11,17]. Maternal mental status and emotional well-being were assessed using 6 questions adapted from the Center for Epidemiological Studies Depression scale [5,18], which is widely used in community-based mental health research. In addition, caregiver-child interactions were assessed using the “Family Care Indicators,” developed by the United Nations Children’s Fund [19], which measure the frequency and type of stimulation and engagement provided by caregivers.

### Data Collection

Once the children were settled after hospital admission and had received initial management, a trained interviewer briefed the caregivers about the project and built rapport. The interviewers were university graduates with previous experience in child health–related research. Informed written consent (signature or fingerprint) was obtained from the caregiver who agreed to participate in the study. Care was taken to organize the questionnaire to minimize the likelihood of caregivers feeling guilty, ashamed, or offended when responding to questions about shaking. For example, caregivers were first informed about common parental habits for disciplining their children. Then they were asked whether they had ever disciplined their children and, if so, how. The conversation was gradually guided to include shaking as one possible disciplinary behavior, after which the interviewer asked whether they had ever shaken their child as a form of discipline. Typical shaking was described as “back and forth” shaking, as used in the CTSPC. If the interviewers were convinced that a shaking incident was being described, additional details were collected. However, “mild” shaking, such as soothing, rocking, or tossing the child up in the air, was not counted as “typical shaking.” The interviews were conducted in a private location in the ward, and the caregivers were offered snacks. One designated health worker or attendant looked after the child during the interview. The collected data were cross-checked by the supervisors on the day of the interview or the following day for any inconsistencies or missing information before data entry.

### Analysis

Data were analyzed using SPSS software for Windows (version 20.0, IBM Corp). A simple descriptive analysis was conducted for this pilot study. Frequency distributions of all the items in the questionnaire were determined. The dichotomous “yes or no” information on whether the child had ever been shaken was calculated by combining the shaking histories reported by the primary caregiver and other family members. Sometimes a child was shaken, either by a caregiver or other family members, in the past year or since birth (if aged <1 year), and these instances were combined to calculate the total number of times the child had been shaken by someone. This variable was not normally distributed. Therefore, the Spearman correlation was conducted separately for the urban (n=520) and the rural (n=280) samples to examine the associations between various sociodemographic and maternal factors and the frequency of shaking the index child. An independent sample 2-tailed *t* test was conducted to compare the populations of the urban and rural areas. Finally, multiple logistic regression was computed to examine the factors associated with shaking as a disciplinary practice with young children. The model considered the following covariates in the analysis: family members (ie, number of persons eating from the same pot), number of children aged younger than 5 years in the household, family income-expenditure deficit, maternal depression, and geographic location (urban and rural). All statistical tests were performed using 2-tailed *t* tests.

### Ethical Considerations

The institutional review board at the International Centre for Diarrhoeal Disease Research, Bangladesh, reviewed and approved this study (2009-007). We obtained verbal informed consent from all parents, and the principles of confidentiality and anonymity were upheld throughout the study. All participants were informed in Bengali about their rights regarding voluntary participation and their freedom to withdraw from the interview at any point during the process.

## Results

### Description of the Population

A total of 800 mother-child dyads were enrolled from both hospitals (Dhaka: n=520, 65%; Matlab: n=280, 35%). Almost all (n=786, 98.3%) of the caregivers were biological mothers of the index children. A significantly higher proportion of fathers in rural areas were engaged in unskilled occupations compared to their urban counterparts (n=34, 10% vs n=45, 18%; *P*=.04). Multimedia Appendix 1 presents the socioeconomic, maternal, and child characteristics of the study populations. In addition, rural families tended to be slightly larger, and the fathers in these areas reported lower household incomes relative to those in urban settings. Across both groups, the mothers generally had a low level of educational attainment, reflecting limited access to formal education in these lower-income communities.

Approximately 15% (n=78) of the urban mothers and 19% (n=53) of the rural mothers completed education beyond high school. Although the mothers in the rural area were significantly better educated than their urban counterparts, they were more likely to be housewives. Approximately 29% (n=151) of the urban fathers and 30% (n=85) of the rural fathers studied beyond high school. Despite the urban-rural differences in living conditions, the monthly income and expenditure balances were not significantly different between the 2 groups. The percentage of mothers with higher depression scores (ie, median >25) was similar in both urban and rural contexts. A higher number of male children were admitted to and enrolled from the rural Matlab hospital than from the urban Dhaka hospital during the study period.

### Shaking Practices in the Communities

In response to the question whether the child had ever been shaken to discipline them by anyone, 51% (n=265) of the respondents in Dhaka and 21% (n=60) of the respondents in Matlab hospital said “yes,” and the percentage was significantly higher (*P*<.001) in the lower-income urban communities of Dhaka compared to rural Matlab. The median number of shaking episodes reported in the past year was 5 (IQR 3-7) in Dhaka and 4 (IQR 2-7) in Matlab.

Responses to the question “How many times in the past year [or since birth if aged <1 year] have you shaken the child?” are illustrated in Multimedia Appendix 2, according to the percentage of children in different age groups who were shaken in Dhaka and Matlab.

### Factors Triggering Shaking

To identify the source of disturbances that triggered shaking behavior, whether originating from the child or the caregiver, we asked specific questions to the caregivers. In most cases, caregivers reported that the shaking was triggered by the child’s behavior: 50.6% (n=134) in Dhaka and 18.3% (n=11) in Matlab. However, 12% (n=32) of the caregivers in Dhaka and 50% (n=30 in Matlab reported that there was no provocation from the child; rather, they were disturbed and stressed for some other familial reasons and expressed their frustrations by shaking the child.

When we asked about specific activities of a child that elicited shaking behavior, caregivers from both Dhaka and Matlab reported that crying and fussing were the most common triggers (n=133, 62.4%) for shaking, particularly in infants approximately aged 6 months (Multimedia Appendix 3). Disruptive behavior of children during feeding (eg, not eating and throwing food) and playing (eg, hitting other children or making a mess) also appeared to be important triggers.

### Persons Involved in Shaking Behavior

In these communities, children’s biological mothers, as the primary caregivers, shook their children the most (Multimedia Appendix 4). Approximately 43% (n=113) of the mothers in urban Dhaka and 60% (n=36) of the mothers in rural Matlab acknowledged that they were the only caregivers who shook the child. Sometimes more than 1 caregiver shook the child (n=77, 29.1% of the cases in Dhaka vs n=4, 6.7% of the cases in Matlab), and in most cases, these caregivers were fathers, grandparents, older siblings, or neighbors, along with the mothers.

A comparatively smaller proportion of biological fathers reported shaking their children: only 8% (n=21) in Dhaka and 5% (n=3) in Matlab. In Dhaka, 20% (n=54) of the children were shaken by nonparent caregivers; among them, 14% (n=37) were siblings, cousins, neighbors, and housemaids, 4% (n=12) were aunts and 2% (n=5) were grandmothers. Similarly, in Matlab, 28% (n=17) of the children were shaken by nonparent caregivers; among them, 10% (n=6) were siblings, 17% (n=10) were aunts, and 2% (n=1) were grandmothers.

### Caregivers’ Beliefs Regarding Child Shaking Behavior

When caregivers were asked about their beliefs regarding shaking, 56.6% (n=85) of the mothers in Dhaka and 28.2% (n=10) of the mothers in Matlab stated that they considered shaking to be harmless for children (Multimedia Appendix 5).

### Factors Associated with Shaking

Finally, to identify the factors associated with shaking as a disciplinary practice in young children, we conducted a multiple logistic regression analysis (Multimedia Appendix 6). The variables included were age and sex of child, parental education (ie, years of schooling), parental occupation, and total number of family members. After adjusting for potential covariates, child age in months was significantly associated with increased odds of being shaken (adjusted odds ratio [AOR] 1.06, 95% CI 1.02-1.10; *P*=.001), showing that older children were more likely to be subjected to this form of discipline. No significant associations were found for child sex, parental occupation, family size, number of children, and parental education. Although male children had higher odds of being shaken compared to female children (AOR 1.13, 95% CI 0.83-1.55; *P*=.44), the association was not statistically significant. Similarly, low-skilled paternal occupation (AOR 1.07, 95% CI 0.75-1.51; *P*=.72) and maternal housewife status (AOR 1.23, 95% CI 0.77-1.98; *P*=.39) were not statistically significant predictors. However, maternal depressive symptoms were positively associated with shaking behavior; each 1-point increase in the depression score was associated with a 3% increase in the odds of shaking (AOR 1.03, 95% CI 1.01-1.04; *P*=.001). In addition, children residing in Dhaka had significantly higher odds (5 times more) of being shaken compared to those in Matlab (AOR 5.32, 95% CI 3.62-7.82; *P*<.001).

## Discussion

### Principal Findings

The practice of shaking as a form of disciplining children, particularly those aged between 6 and 12 months, was found to be unexpectedly high in lower-income rural and urban households. Around 27% (n=214) of the primary caregivers (mothers) of the children surveyed had practiced it. This study was designed to document the occurrence of shaking and not its severity.

One possible explanation for the high prevalence of caregiver-reported shaking behavior among those aged between 6 and 12 months is the increased caregiving demands during this developmental period, including heightened crying, mobility, and feeding difficulties. Children require more attention and care during this critical time as they transition to complementary feeding; learn new developmental skills, such as motor activities (eg, crawling and sitting); and begin to explore their surroundings. Moreover, infants at this age experience frequent illnesses (eg, diarrhea, respiratory infections, and fever) due to the waning of maternally acquired immunity and increased exposure to infectious agents. Consequently, caregivers become increasingly anxious and stressed, which can contribute to “shaking” as a response to perceived child distress or behavioral challenges. Unlike studies from high-income countries, shaking behavior does not appear to be prevalent during early infancy (ie, aged <6 months). One explanation could be the culture and social structure that exists between neighboring families in Bangladesh. Families in these communities tend to live closely together, offer mutual support, and form very intimate bonds. Such connections may serve as a buffer against caregiver stress during the first months of childcare. In rural Matlab, 3 to 5 households share a common courtyard [20], and in Dhaka slums, as many as 8 to 10 individuals share a 1-room dwelling [21]. Thus, there are several household members available to help with childcare during the early months.

The peak prevalence of shaking is similar in urban and rural households, although the practice was significantly higher in the slums of Dhaka city. Both groups have experienced extreme poverty, but there are important differences as well. Urban mothers are more likely to be wage earners and have a higher level of education. Unlike in other LMICs, where maternal education had a negative correlation with shaking behavior [22], we found no significant association between maternal education and shaking practices, although it was higher in urban mothers. However, maternal depressive symptoms showed a significant association with shaking practices for disciplining [23]. This indicates the importance of maternal mental well-being as the primary caregiver.

Similar to other studies, we found crying and fussing to be common triggering factors for shaking, particularly in young children. However, the timing of crying is outside the normal cry curve, which peaks at around 3 months of age in high-income countries [24]. Among 33 infants aged younger than 3 months, only 4 (12%) in Dhaka and none in Matlab experienced shaking.

We also tried to assess the mental status of the caregivers during shaking by asking questions about how frustrated or angry they were and why. Approximately 50% (n=162) of the mothers reported that they were disturbed or frustrated at the time of their last shaking episode due to different reasons (eg, a quarrel with their husband or mother-in-law). In our study, depression was positively correlated with the shaking behavior of mothers.

According to one study in Ethiopia, female primary caregivers were significantly associated with a higher use of harsh physical punishment compared to male caregivers [23]. We found female caregivers (eg, mothers, aunts, sisters, and grandmothers) to be the main perpetrators of shaking behavior. This contrasts with studies published from high-income countries, where in 65% to 90% of the cases, young male individuals—either the baby’s father or the mother’s boyfriend—are the main perpetrators [25]. This could be due to the cultural norms in this part of the world, where mothers and other female relatives are expected to look after children at an early age.

However, consistent with studies from many other countries, half (n=85, 56.6%) of the mothers in Dhaka hospital and a third (n=10, 28.2 %) of the mothers in Matlab believed that infant shaking is harmless. Interestingly, when we interviewed mothers to learn how they discipline their children, not a single mother spontaneously mentioned shaking as a disciplining behavior. However, when asked specifically whether they shook their child for discipline, they reported that they did so sometimes. This indicates that, culturally, shaking is not considered a very dangerous or cruel behavior in child-rearing in Bangladesh.

In general, this pilot study aimed to explore the prevalence of shaking behavior among caregivers toward their young children residing in lower-income communities as well as the possible biological and sociodemographic factors associated with it. We collected information from the aforementioned 2 popular diarrhea hospitals because a large number of people from lower-income communities with mild to moderate illnesses are admitted to these hospitals. Therefore, selecting cases from these hospitals allowed us to estimate the prevalence of shaking in lower-income, stressed, and vulnerable communities of urban and rural Bangladesh.

### Limitations

Our main limitation was the selection of a biased sample of mothers of children who were hospitalized with illness. To minimize this, we included only the mothers of children with less severe illness who were admitted to longer-stay units, so that after settling their children, they could talk to us. Most of the reported studies on infant shaking interviewed mothers of children diagnosed with SBS and thus were easier to interpret. Here, based on the mother’s report, we do not know how severe the shaking was and whether it impacted the child. The report of maternal depression was also confounded by the concurrent illnesses of the children.

Apart from these limitations, this quick exploratory study on 720 participants revealed the context of infant-shaking behavior in an LMIC such as Bangladesh. The key observations were that shaking behavior exists in lower-income communities in Bangladesh and is alarmingly high, but the patterns are different from those reported in high-income countries. The age range for children who were shaken is different from that in the Western context, as shaking occurs when children are slightly older. Although similar to other countries, crying and fussing triggered shaking behavior in most cases; this does not fall within the normal crying curve of infants aged 2 to 3 months. Mothers are the main perpetrators of infant shaking because of their frequent contact with children, and in many cases, they are unaware of the hazardous outcomes.

Furthermore, qualitative and quantitative studies are needed to determine the actual burden of this problem. In Bangladesh, child abuse and neglect are becoming a growing concern [26]. A screening program on 2576 children in Bangladesh showed that up to 70% of the children are living with mild to moderate disabilities [27]. It is also important to examine the relationship between early shaking and future child development in low-income countries to understand the long-term consequences of infant shaking and the resulting social and public health burden.

### Comparison with Prior Work

There has been no study conducted in Bangladesh with which we can compare our results.

### Conclusions

This study provides insights into the differences in disciplinary acts experienced by children from their caregivers in 2 types of communities in Bangladesh. We recommend more research on this topic, considering cultural beliefs or social practices. It is very important to examine the association between shaking behavior and child development. In addition, we found that maternal depression was correlated with certain disciplining practices, which, as we hypothesize, may affect child development. Children living in lower-income urban communities are more prone to these practices. However, the long-term effects of disciplining children through shaking practices are still unknown. We need to help parents understand how such behavior impacts their children. As the world shifts into a new era, some social aspects of raising children need to be revised for the betterment of society.

## Appendix

Multimedia Appendix 1 Socioeconomic and child characteristics of the population studied.

Multimedia Appendix 2 Percentage of children ever shaken by caregivers, by age group, in Dhaka and Matlab hospitals.

Multimedia Appendix 3 Triggers for shaking behavior (child’s activities).

Multimedia Appendix 4 Child-shaking by caregivers in Dhaka and Matlab.

Multimedia Appendix 5 Mothers’ perception of shaking.

Multimedia Appendix 6 Factors linked to shaking as discipline among young children.

## Abbreviations

AHT: abusive head trauma
AOR: adjusted odds ratio
CTSPC: Parent-Child Conflict Tactics Scale
LMIC: low- and middle-income country
SBS: shaken baby syndrome
