# Supplementary material for: The Practice of Shaking in Disciplining Young Children in Lower-Income Communities of Bangladesh: Cross-Sectional Exploratory Study
Source: JMIR Pediatr Parent. 2025 Oct 14;8:e64474. doi: 10.2196/64474 (PMC12569487; doi:10.2196/64474)
Supplement: Multimedia Appendix 1 [file pediatrics_v8i1e64474_app1.docx]

**Multimedia Appendix 1. Socioeconomic and Child Characteristics of the population studied**

| Socio-economic factors | | | | Dhaka Hospital  [n=520] | Matlab Hospital  [n=280] |
| --- | --- | --- | --- | --- | --- |
| Income-expenditure balance | | % of constant deficit [n=102] | | 14.6 | 7.5 |
|  |  | % of occasional deficit [n=169] | | 19.3 | 25.0 |
|  |  | % balanced [n=243] | | 24.7 | 41.4 |
|  |  | % surplus[n=286] | | 41.4 | 26.1 |
| # Family member [median, range] | | | | 5 [4, 6] | 6 [4 to 8] |
| # of <5-year-old children [median, range] | | | | 3 [1, 4] | 4 [2, 6] |
| Father’s education [% >10 year] | | | | 29 | 30 |
| Father’s years of education [mean ±SD] | | | | 6.9±4.6 | 7.3±4.1 |
| Mother’s education [% >10 year] | | | | 15% | 19% |
| Mother’s years of education [mean ±SD] | | | | 5.7±4.0 | 7.1±3.6 |
| Mother’s occupation [% housewives] | | | | 84 | 96 |
| Father’s occupation | % unskilled job [n=100] | | | 10 | 18 |
|  | % low-skilled job [n=313] | | | 41 | 35 |
|  | % regular & high skilled job [n=387] | | | 49 | 47 |
| Maternal depression score [range 0-42, mean ± SD] | | | | 18.7±2.7 | 19.0±3.1 |
| Maternal depression score [% high score >25] | | | | 26 | 29 |
| Children’s characteristics | | | | | |
| The mean age of the child in months | | |  | 8.7±4.1 | 11.3±4.9 |
| Birth order [Median, range] | | |  | 2 [1, 2] | 2 [2, 4] |
| Male children [%] | | |  | 57% | 64% |
