# Supplementary material for: The Practice of Shaking in Disciplining Young Children in Lower-Income Communities of Bangladesh: Cross-Sectional Exploratory Study
Source: JMIR Pediatr Parent. 2025 Oct 14;8:e64474. doi: 10.2196/64474 (PMC12569487; doi:10.2196/64474)
Supplement: Multimedia Appendix 2 [file pediatrics_v8i1e64474_app2.docx]

**Multimedia Appendix 2.** Percentage of children ever shaken by caregivers, by age group, in Dhaka and Matlab hospitals

|  | | Children, n (%) |
| --- | --- | --- |
| **Children in Dhaka hospital by age group (mo)** | | |
|  | <3 | 4 (1.5%) |
|  | 3-6 | 45 (17.0% |
|  | 6-9 | 90 (34.0%) |
|  | 9-12 | 71 (26.8%) |
|  | 12-15 | 36 (13.6%) |
|  | >15 | 19 (7.22%) |
| **Children in Matlab hospital by age group (mo)** | | |
|  | <3 | 0 (0.0%) |
|  | 3-6 | 4 (6.7%) |
|  | 6-9 | 11 (18.3%) |
|  | 9-12 | 21 (35.0%) |
|  | 12-15 | 11 (18.3%) |
|  | >15 | 13 (21.7%) |
