# Supplementary material for: The Practice of Shaking in Disciplining Young Children in Lower-Income Communities of Bangladesh: Cross-Sectional Exploratory Study
Source: JMIR Pediatr Parent. 2025 Oct 14;8:e64474. doi: 10.2196/64474 (PMC12569487; doi:10.2196/64474)
Supplement: Multimedia Appendix 3 [file pediatrics_v8i1e64474_app3.docx]

**Multimedia Appendix 3.** Triggers for shaking behavior (child’s activities)

|  | n | % |
| --- | --- | --- |
| **Dhaka [n=520]** | | |
| **Total reports of child-shaking in Dhaka** | **265** | **51** |
| - Triggering factors reported in shaken children | 174 | 65.7 |
| - Triggering factors not known in shaken children | 91 | 34.3 |
| Child ‘s activities that triggered shaking | | |
| - Crying | 88 | 50.6 |
| - Becoming naughty during playing | 26 | 14.9 |
| - Fussing | 19 | 10.9 |
| - Laughing | 11 | 6.3 |
| - Refusing to eat | 11 | 6.3 |
| - Disturbing while pooping | 5 | 2.9 |
| - Disturbing during eating food | 2 | 1.2 |
| - Making a mess | 1 | 0.6 |
| - Other causes [vomiting, hitting other babies, eating wall paint, inserting finger to electric switch etc.] | 11 | 6.3 |
| **Matlab [n=280]** | | |
| **Total reports of child-shaking in Matlab** | **52** | **19** |
| - Triggering factors reported | 39 | 75.0 |
| - Triggering factors not known | 13 | 25.0 |
| Child ‘s activities that triggered shaking | | |
| - Fussing | 14 | 35.9 |
| - Crying | 12 | 30.8 |
| - Refusing to eat | 5 | 12.82% |
| - Making a mess | 5 | 12.82% |
| - Having a temper tantrum | 1 | 2.56% |
| - Other causes | 2 | 5.13% |
