# Supplementary material for: The Practice of Shaking in Disciplining Young Children in Lower-Income Communities of Bangladesh: Cross-Sectional Exploratory Study
Source: JMIR Pediatr Parent. 2025 Oct 14;8:e64474. doi: 10.2196/64474 (PMC12569487; doi:10.2196/64474)
Supplement: Multimedia Appendix 4 [file pediatrics_v8i1e64474_app4.docx]

**Multimedia Appendix 4.** Child-shaking caregivers in Dhaka and Matlab

| **Caregiver shook children in Dhaka** | n (%) |
| --- | --- |
| Mothers | 113 (42.6%) |
| Fathers | 21 (7.9%) |
| Grandma | 5 (1.9%) |
| Brothers | 10 (3.8%) |
| Sisters | 12 (4.5%) |
| Aunt/Uncle | 12 (4.5%) |
| Cousins/Neighbors | 15 (5.7%) |
| >2 persons including mothers | 77 (29.1%) |
| **Caregiver shook children in Matlab** | n (%) |
| Mothers | 36 (60.0%) |
| Fathers | 3 (5.0%) |
| Grandma | 1 (1.7%) |
| Brothers | 2 (3.3%) |
| Sisters | 2 (3.3%) |
| Aunt/Uncle | 10 (16.7%) |
| Cousins/Neighbors | 2 (3.3%) |
| >2 persons including mothers | 4 (6.7%) |
