# Supplementary material for: The Practice of Shaking in Disciplining Young Children in Lower-Income Communities of Bangladesh: Cross-Sectional Exploratory Study
Source: JMIR Pediatr Parent. 2025 Oct 14;8:e64474. doi: 10.2196/64474 (PMC12569487; doi:10.2196/64474)
Supplement: Multimedia Appendix 5 [file pediatrics_v8i1e64474_app5.docx]

**Multimedia Appendix 5. Mothers’ perception of shaking**

| Whether the child is hurt when mothers shake him/her | | |
| --- | --- | --- |
|  | Dhaka Hosp | Matlab Hosp |
| Not | 8% | 2.6% |
| Probably did not hurt | 48.6% | 25.6% |
| Hurt the baby a little | 25.1% | 56.4% |
| Hurt the baby a lot | 17.7% | 15.4% |
| Don’t know | 0.6% | ------- |
| Whether the child is hurt when others shook him/her | | |
| Not | 6.6% | 20.8% |
| Probably did not hurt | 37.5% | 16.7% |
| Hurt the baby a little | 27% | 50% |
| Hurt the baby a lot | 28.3% | 12.5% |
