# Supplementary material for: The Practice of Shaking in Disciplining Young Children in Lower-Income Communities of Bangladesh: Cross-Sectional Exploratory Study
Source: JMIR Pediatr Parent. 2025 Oct 14;8:e64474. doi: 10.2196/64474 (PMC12569487; doi:10.2196/64474)
Supplement: Multimedia Appendix 6 [file pediatrics_v8i1e64474_app6.docx]

**Multimedia Appendix 6.** Factors linked to shaking as discipline among young children

| **Characteristics** | **Shaking in Discipline*** | |
| --- | --- | --- |
|  | **AOR (95% CI)** | **p-value** |
| **Age (Months)** | 1.06 (1.02, 1.10) | 0.001 |
| **Sex** |  |  |
| Female | — |  |
| Male | 1.13 (0.83, 1.55) | 0.435 |
| **Father occupation** |  |  |
| High Skill | — |  |
| Low Skill | 1.07 (0.75, 1.51) | 0.717 |
| **Mother occupation** |  |  |
| Working | — |  |
| Housewife | 1.23 (0.77, 1.98) | 0.385 |
| **Family member** |  |  |
| >5 members | — |  |
| ≤= 5 members | 0.98 (0.71, 1.34) | 0.878 |
| **Number of under-five children in the house** |  |  |
| >3 children | — |  |
| ≤= 3 children | 0.77 (0.56, 1.05) | 0.102 |
| **Father education** | 1.00 (0.95, 1.04) | 0.831 |
| **Mother education** | 1.04 (0.98, 1.09) | 0.213 |
| **Income** | 1.03 (0.88, 1.21) | 0.731 |
| **Depression total** | 1.03 (1.01, 1.04) | 0.001 |
| **Place** |  |  |
| Matlab | — |  |
| Dhaka | 5.32 (3.62, 7.82) | <0.001 |
| * No shaking is the reference category, AOR= Adjusted Odds Ratio, CI = Confidence Interval | | |
